# Is the presence of medical trainees associated with increased mortality with weekend admission?

**DOI:** 10.1186/1472-6920-14-4

**Published:** 2014-01-08

**Authors:** Rocco Ricciardi, Jason Nelson, Patricia L Roberts, Peter W Marcello, Thomas E Read, David J Schoetz

**Affiliations:** 1 Department of Colon and Rectal Surgery, Lahey Clinic, Tufts University, 41 Mall Rd, Burlington, MA 01805, USA

**Keywords:** Mortality, Weekend, Trainee, Non-elective

## Abstract

**Background:**

Several studies have demonstrated increased inhospital mortality following weekend admission. We hypothesized that the presence of resident trainees reduces the weekend mortality trends.

**Methods:**

We identified all patients with a non-elective hospital admission from 1/1/2003 through 12/31/2008. We abstracted vital status on discharge and calculated the Charlson comorbidity score for all inpatients. We compared odds of inpatient mortality following non-elective admission on a weekend day as compared to a weekday, while considering diagnosis, patient characteristics, comorbidity, hospital factors, and care at hospitals with resident trainees.

**Results:**

Data were available for 48,253,968 patient discharges during the six-year study period. The relative risk of mortality was 15% higher following weekend admission as compared to weekday admission. After adjusting for diagnosis, age, sex, race, income level, payer, comorbidity, and weekend admission the overall odds of mortality was higher for patients in hospitals with fewer nurses and staff physicians. Mortality following a weekend admission for patients admitted to a hospital with resident trainees was significantly higher (17%) than hospitals with no resident trainees (p < 0.001).

**Conclusions:**

Low staffing levels of nurses and physicians significantly impact mortality on weekends following non-elective admission. Conversely, patients admitted to hospitals with more resident trainees had significantly higher mortality following a weekend admission.

## Background

A growing number of studies have demonstrated increased mortality on weekends for patients suffering from several urgent medical and surgical diagnoses [1,2]. Increasingly, there is evidence suggesting that patient care, especially for those with time-sensitive conditions [3], is compromised during weekends and that patients become unnecessarily vulnerable on weekends. No clear explanation for this mortality difference exists; however a number of potential factors should be considered. Factors influencing excess weekend mortality may include reduced staff levels, wide physician cross-coverage, and fewer specialized diagnostic, procedural, and treatment options on the weekend [3].

One of the greatest concerns regarding weekend staffing is that fewer nurses and staff physicians are available, while less-experienced caregivers provide the bulk of care. In addition, resident physicians or trainees often provide many urgent care services on nights and weekends. Given the often front line role that resident physicians have in patient care on weekends, we performed an analysis to delineate the potential effect of resident trainees on disparate outcomes based on day of admission. Our hypothesis was that resident trainees act as front line caregivers and thus, hospitals with residency programs may experience a reduced overall mortality following weekend admission. Low staffing levels and a lack of resident trainees may lead to care that is untimely and error prone resulting in higher mortality following weekend admission.

## Methods

### Data source

We obtained all-payer discharge data from January 1, 2003, through December 31, 2008, via the Nationwide Inpatient Sample (NIS) of the Healthcare Cost and Utilization Project of the Agency for Healthcare Research and Quality. The NIS—the largest source of all-payer hospital discharge information in the United States—contains data from approximately 7 million to 8 million hospital stays per year in 1000 hospitals in over 30 states [4]. The hospitals sampled can vary from year to year but the sample approximates 20% of US community hospitals including large university hospitals and smaller regional facilities. The database provides information regarding patient demographics, socioeconomic factors, admission profiles, hospital profiles, state codes, discharge diagnoses, procedure codes, total charges, and vital status at hospital discharge. Along with other hospital discharge databases, the NIS has been used to review trends in surgical care and outcomes [5], volume outcome relationships [6], and disparities in care [7]. A data use agreement is held by the Agency for Healthcare Research and Quality, and our study was considered exempt by the Lahey Clinic Institutional Review Board.

The American Hospital Association (AHA) Annual Survey of Hospitals database was obtained in order to determine facility structural characteristics, service lines, staffing, and the presence of resident trainees at each hospital. The AHA database contains hospital-specific information on over 6000 hospitals and over 450 health-care systems, including 700 data elements [8]. The purpose of the AHA database is to generate a comprehensive and inclusive overview of hospitals while permitting the tracking of hospital performance over time. AHA data have been extensively used to study hospital-based outcomes [9], hospital policies [10], and reimbursement [11].

### Study population

All patients discharged during the time frame sampled were included (both medical and surgical patients). We used the elective variable to exclude all patients with an admission for elective reasons and included only those patients with nonelective admission [4]. Thus, patients with emergency and urgent indications for admission were included in our study.

### Admission day

The data set permits identification of admission day as a weekend or weekday. We recorded this variable as admitted during a weekend (i.e., Saturday or Sunday) or a weekday (i.e., Monday through Friday) [1,4].

### Covariates

Our analysis adjusted for the following covariates: age, sex, race, income level, payer, major diagnostic categories (subgroupings of diagnosis-related groups),^1^ and the Charlson comorbidity index score. Age was included as a continuous variable. Sex was entered as a dichotomous variable. Race was divided into white, black, Hispanic, Asian or Pacific Islander, Native American, or other. Income level was categorized into quartiles per estimated median household income of residents in the patient’s zip code [4]. The median income quartiles are classified as follows: $0 to $38 999, $39 000 to $47 999, $48 000 to $62 999, and $63 000 or more [4].

Payer was recorded as follows: Medicare, Medicaid, private including health maintenance organization, self-pay, no charge, or other [4]. Major diagnostic categories were used to adjust for diagnoses and reflect larger groupings of diagnostic-related groups made available in the provided data set and downloadable for review from the US Department of Health and Human Services, Centers for Medicare and Medicaid Services [12]. Major diagnostic categories have been used to evaluate hospitalization risk [13], mortality risk [14], and other outcomes [15]. We also evaluated comorbidity with the Deyo modification of the Charlson comorbidity index [16]. Briefly, we ascertained the presence of 17 comorbid conditions and then weighted them according to the original report. An elevated Charlson comorbidity index score has been demonstrated to correlate with higher mortality rate [17].

Hospital bed size categories were obtained from the American Hospital Association Annual Survey of Hospitals and based on the number of short-term acute care beds.

### Staffing

Staffing levels were obtained from the American Hospital Association Annual Survey of Hospitals [8]. We analyzed the role of full-time registered nurses and full-time physicians on mortality by developing ratios of either nurse or physician per hospital bed. We categorized these two variables into tertiles, low, medium, or high.

### Presence of resident trainees

The teaching status of the hospital was obtained from the American Hospital Association Annual Survey of Hospitals [8]. Presence of resident trainees was categorized into tertiles. Given that half of all facilities had no residents, this was the lowest tertile. The middle tertile included 1–26 resident trainees. The highest tertile included greater than or equal to 27 resident trainees.

### Outcome

The data set permits identification of vital status at the time of discharge. The variable is coded as died during hospitalization or did not die during hospitalization. Deaths that occurred after discharge are not identifiable from our data set [4].

### Statistical analysis

Statistical analyses were performed using SAS statistical software, version 9.2 (SAS Institute Inc, Cary, North Carolina). We analyzed univariate associations with patient admission day (weekend vs. weekday) using t tests for continuous variables and *χ*2 tests for categorical variables. Results were considered statistically significant at p < 0.05, and all statistical tests were 2-tailed. We included all covariates in our regression model. The analyses were conducted with and without missing data. To confirm results, we performed imputation of missing data using the multiple imputation procedure from SAS Institute Inc [18]. Imputation substitutes missing values with plausible values that characterize the uncertainty regarding the missing data [19,20]. This process results in valid statistical inferences that properly reflect the uncertainty due to missing values, for example, confidence intervals with the correct probability coverage. The multiply imputed dataset was then analyzed by using standard logistic regression for the complete data.

We tested for interactions between staffing levels, resident trainees and admission day on mortality in the regression analysis.

## Results

### Cohort

Data were available for 48,253,968 patient discharges during the six-year study period, of which 26,038,921 were non-elective. Demographics, patient characteristics, and comorbidity are listed in Table 1 in relation to day of admission. In addition, Table 2 lists the hospital characteristics, staffing levels, and other AHA variables included in our analysis.

**Table 1:** Demographics of patients admitted to the hospital for non-elective indications on a weekday compared with the weekends

**Demographics**	**Weekend**	**Weekday**	**All patients**
	**(N = 5,998,016)**	**(N = 20,053,759)**	**(N = 26,051,775)**
Age, mean (SD)	47.9 (29.6)	46.9 (29.7)	47.1 (29.6)
Sex			
Male	2,651,234 (23.3)	8,747,217 (76.7)	11,398,451
Female	3,321,072 (22.8)	11,228,425 (77.2)	14,549,497
Missing	25,710 (24.8)	78,117 (75.2)	103,827
Race			
White	3,017,636 (22.7)	10,304,064 (77.3)	13,321,700
Asian	139,398 (23.6)	452,512 (76.4)	591,910
Black	676,910 (23.3)	2,232,936 (76.7)	2,909,846
Hispanic	670,770 (23.3)	2,211,791 (76.7)	2,882,561
Native American	24,399 (23.4)	79,707 (76.6)	104,106
Other	142,420 (23.0)	478,022 (77.0)	620,442
Missing	1,326,483 (23.6)	4,294,727 (76.4)	5,621,210
Median Household Income			
0-25th Percentile	1,550,655 (23.1)	5,171,693 (76.9)	6,722,348
26-50th Percentile	1,477,013 (23.1)	4,919,398 (76.9)	6,396,411
51-75th Percentile	1,407,992 (23.0)	4,708,169 (77.0)	6,116,161
76-100th Percentile	1,402,397 (22.9)	4,723,520 (77.1)	6,125,917
Missing	159,959 (23.2)	530,979 (76.8)	690,938
Primary Payer			
Private	1,916,698 (22.3)	6,693,914 (77.7)	8,610,612
Medicaid	1,185,764 (22.8)	4,018,221 (77.2)	5,203,985
Medicare	2,323,860 (23.5)	7,560,323 (76.5)	9,884,183
None	38,416 (25.0)	115,206 (75.0)	153,622
Other	183,121 (22.4)	635,129 (77.6)	818,250
Self-Pay	342,981 (25.4)	1,007,693 (74.6)	1,350,674
Missing	7,176 (23.6)	23,273 (76.4)	30,449
Comorbidity Score, mean (SD)	1.1 (1.7)	1.1 (1.7)	1.1 (1.7)
MDC			
Nervous System	397,624 (25.2)	1,178,110 (74.8)	1,575,734
Alcohol/Drug Use	73,234 (23.1)	244,275 (76.9)	317,509
Burns	6,909 (29.0)	16,888 (71.0)	23,797
Circulatory	991,071 (22.3)	3,455,504 (77.7)	4,446,575
Digestive	609,727 (24.4)	1,888,117 (75.6)	2,497,844
Endocrine System	188,972 (22.9)	636,572 (77.1)	825,544
Eyes	10,662 (26.4)	29,663 (73.6)	40,325
Female Reproductive System	27,132 (16.0)	142,867 (84.0)	169,999
Hepatobiliary	194,396 (24.2)	609,474 (75.8)	803,870
HIV	17,490 (22.3)	61,097 (77.7)	78,587
Immunologic	70,411 (21.3)	259,794 (78.7)	330,205
Infectious	182,957 (25.2)	541,981 (74.8)	724,938
Injuries/Poisons	116,601 (27.4)	309,638 (72.6)	426,239
Kidneys	250,741 (23.8)	804,607 (76.2)	1,055,348
Male Reproductive System	12,086 (20.6)	46,500 (79.4)	58,586
Mental Health	213,546 (19.6)	875,088 (80.4)	1,088,634
Musculoskeletal	329,252 (24.6)	1,007,043 (75.4)	1,336,295
Myeloproliferative	20,791 (14.3)	124,352 (85.7)	145,143
Neonatal	793,403 (21.0)	2,984,827 (79.0)	3,778,230
Other Health Factors	33,873 (15.5)	184,566 (84.5)	218,439
Otorhinolaryngology	75,273 (25.7)	218,042 (74.3)	293,315
Pregnancy And Childbirth	478,320 (21.6)	1,736,923 (78.4)	2,215,243
Pre-Major Diagnostic Category	3,621 (27.2)	9,677 (72.8)	13,298
Respiratory	731,704 (25.2)	2,175,813 (74.8)	2,907,517
Skin Or Breast	145,317 (23.6)	470,145 (76.4)	615,462
Trauma	22,903 (35.2)	42,196 (64.8)	65,099

Proportions reported in parenthesis unless otherwise noted.

Data are presented as number of discharges (percent) unless otherwise indicated. All comparisons by admission day are statistically significant, p < 0.001.

**Table 2:** Hospital characteristics of patients admitted to the hospital for non-elective indications on a weekday compared with the weekends

**Hospital characteristic**	**Weekend**	**Weekday**	**All patients**
Hospital Control			
Not-For-Profit, Nongovernment	4,458,164 (23.1)	14,816,595 (76.9)	19,274,759
Government	837,958 (23.0)	2,811,631 (77.0)	3,649,589
For-Profit, Investor Owned	701,894 (22.4)	2,425,533 (77.6)	3,127,427
Region			
Northeast	1,621,325 (22.6)	5,545,056 (77.4)	7,166,381
Midwest	954,215 (23.2)	3,156,368 (76.8)	4,110,583
South	1,791,365 (22.8)	6,079,594 (77.2)	7,870,959
West	1,631,111 (23.6)	5,272,741 (76.4)	6,903,852
Urbanicity			
Urban	5,803,138 (23.0)	19,403,387 (77.0)	25,206,525
Rural	191,618 (23.1)	639,070 (76.9)	830,688
Missing	3,260 (22.4)	11,302 (77.6)	14,562
Critical Access			
Yes	127,335 (23.5)	413,869 (76.5)	541,204
No	5,829,471 (23.0)	19,507,489 (77.0)	25,336,960
Missing	41,210 (23.7)	132,401 (76.3)	173,611
Rural Referral Center			
Yes	402,736 (23.1)	1,341,355 (76.9)	1,744,091
No	5,569,464 (23.0)	18,631,686 (77.0)	24,201,150
Missing	25,816 (24.2)	80,718 (75.8)	106,534
Networked			
Yes	2,059,136 (22.8)	6,954,919 (77.2)	9,014,055
No	3,312,180 (23.1)	11,025,023 (76.9)	14,337,203
Missing	626,700 (23.2)	2,073,817 (76.8)	2,700,517
Medical Surgical Intensive Care			
Yes	5,337,106 (23.0)	17,855,395 (77.0)	23,192,501
No	361,847 (23.1)	1,205,097 (76.9)	1,566,944
Missing	299,063 (23.1)	993,267 (76.9)	1,292,330
Hospice Program			
Yes	1,801,819 (23.1)	5,984,198 (76.9)	7,786,017
No	3,831,348 (22.9)	12,878,741 (77.1)	16,710,089
Missing	364,849 (23.5)	1,190,820 (76.5)	1,555,669
Cardiac Intensive Care			
Yes	3,866,696 (23.0)	12,975,259 (77.0)	16,841,955
No	1,792,240 (23.2)	5,946,583 (76.8)	7,738,823
Missing	339,080 (23.1)	1,131,917 (76.9)	1,470,997
Pediatric Hospital			
Yes	4,187,982 (23.0)	14,007,815 (77.0)	18,195,797
No	1,460,456 (23.0)	4,887,722 (77.0)	6,348,178
Missing	349,578 (23.2)	1,158,222 (76.8)	1,507,800
Neonatal Intensive Care Unit			
Yes	3,023,448 (23.0)	10,147,156 (77.0)	13,170,604
No	2,619,397 (23.1)	8,732,575 (76.9)	11,351,972
Missing	355,171 (23.2)	1,174,028 (76.8)	1,529,199
Skilled Nursing Care			
Yes	1,540,309 (23.1)	5,141,015 (76.9)	6,681,324
No	4,087,411 (23.0)	13,707,285 (77.0)	17,794,696
Missing	370,296 (23.5)	1,205,459 (76.5)	1,575,755
JCAHO Accreditation			
Yes	5,621,862 (23.0)	18,819,411 (77.0)	24,441,273
No	376,154 (23.4)	1,234,348 (76.6)	1,610,502
Missing	0	0	0
ACS Cancer Program			
Yes	3,542,482 (23.0)	11,860,791 (77.0)	15,403,273
No	2,455,534 (23.1)	8,192,968 (76.9)	10,648,502
Missing	0	0	0
Radiology, Photon Emission			
Yes	3,711,150 (22.9)	12,462,701 (77.1)	16,173,851
No	1,890,070 (23.1)	6,291,413 (76.9)	8,181,483
Missing	396,796 (23.4)	1,299,645 (76.6)	1,696,441
Radiology, CT Scan			
Yes	4,065,915 (23.0)	13,630,554 (77.0)	17,696,469
No	1,546,023 (23.1)	5,133,106 (76.9)	6,679,129
Missing	386,078 (23.0)	1,290,099 (77.0)	1,676,177
Radiology, PET Scan			
Yes	2,200,507 (22.8)	7,437,175 (77.2)	9,637,682
No	3,416,652 (23.1)	11,381,603 (76.9)	14,798,255
Missing	380,857 (23.6)	1,234,981 (76.4)	1,615,838
Medical Beds			
0-20	253,097 (23.1)	844,804 (76.9)	1,097,901
21-74	779,721 (23.0)	2,603,130 (77.0)	3,382,851
≥74	4,246,857 (23.0)	14,227,412 (77.0)	18,474,269
Missing	718,341 (23.2)	2,378,413 (76.8)	3,096,754
Total Hospital Beds			
0-53	285,873 (23.3)	942,890 (76.7)	1,228,763
54-157	935,537 (23.1)	3,109,974 (76.9)	4,045,511
≥157	4,776,606 (23.0)	16,000,895 (77.0)	20,777,501
Missing	0	0	0
Pediatric Beds			
0	1,652,963 (23.0)	5,523,949 (77.0)	7,176,912
1-15	1,766,364 (23.1)	5,872,580 (76.9)	7,638,944
≥16	1,860,348 (22.9)	6,278,817 (77.1)	8,139,165
Missing	718,341 (23.2)	2,378,413 (76.8)	3,096,754
Medical & Dental Trainees			
0	2,911,505 (23.2)	9,646,519 (76.8)	12,558,024
1-26	1,250,566 (23.1)	4,158,208 (76.9)	5,408,774
≥27	1,835,945 (22.7)	6,249,032 (77.3)	8,084,977
Missing	0	0	
Physicians/Hospital Bed			
0-0.007	1,710,633 (23.1)	5,705,164 (76.9)	7,415,797
0.007-0.067	1,686,461 (23.0)	5,654,953 (77.0)	7,341,414
≥0.067	2,526,572 (23.0)	8,445,660 (77.0)	10,972,232
Missing	74,350 (23.1)	247,982 (76.9)	322,332
Nurses/Hospital Bed			
0-0.75	260,294 (23.0)	873,025 (77.0)	1,133,319
0.75-1.359	1,944,929 (22.9)	6,544,395 (77.1)	8,489,324
≥1.359	3,718,443 (23.1)	12,388,357 (76.9)	16,106,800
Missing	74,350 (23.1)	247,982 (76.9)	322,332

Proportions reported in parenthesis unless otherwise noted.

Data are presented as number of discharges (percent) unless otherwise indicated. All comparisons by admission day are statistically significant, p < 0.001, except for urbanicity (p-value = 0.1195).

### Univariate analysis

**Figure 1:**
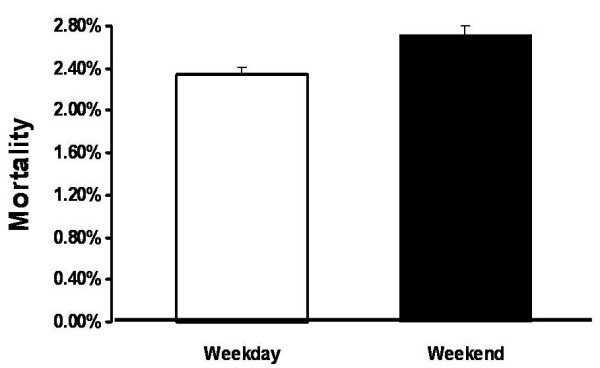
**The risk of mortality on the weekend as compared to a weekday.** Chi-square analysis for significance.

The relative risk of mortality was15% higher on the weekend as compared to a weekday (Figure 1). Patients admitted on the weekend were on average older (47.9 vs. 46.9 years) than those admitted during a weekday. Males were more likely to be admitted on a weekend than females (23.3% vs. 22.8%). Whites were less likely to be admitted on a weekend than all other racial groups. Lower income categories were more likely to be admitted on a weekend than the highest quartile group. Self-paying and patients without health insurance were more likely to be admitted on the weekend (25.4% and 25.0%, respectively) than patients with other primary methods of payment. On average, patients admitted during the weekend had a higher comorbidity score (Tables 1 and 2).

### Multivariate analysis

After adjusting for diagnosis, age, sex, race, income level, payer, comorbidity, and weekend admission the overall odds of mortality was higher for patients in hospitals with fewer nurses and staff physicians. Conversely, hospitals with more physicians or nurses per hospital bed were associated with a 10% reduction in mortality after a weekend admission (Odds Ratio = 0.9: CI 0.9-0.9). In addition, mortality was higher for patients in hospitals with more beds (Odds Ratio = 1.1: CI 1.0-1.1) and more resident trainees (Odds Ratio = 1.05: CI 1.04-1.07) (Table 3).

**Table 3:** Multivariate analysis of mortality for non-elective admissions

**Characteristic**	**Odds ratio**	**Confidence interval**	**p value**
**Age**	1.04	1.04-1.04	0.0001
**Sex**			
**Male**	Reference		
**Female**	0.9	0.8-0.9	0.0001
**Race**			
**White**	Reference		
**Asian**	1.0	1.0-1.0	0.6
**Black**	1.0	1.0-1.0	0.3
**Hispanic**	0.9	0.9-0.9	0.0001
**Native American**	1.0	0.9-1.0	0.4
**Other**	1.1	1.1-1.1	0.0001
**Median Household Income**			
**0-25th Percentile**	Reference		
**26-50th Percentile**	0.98	0.97-0.99	0.0001
**51-75th Percentile**	0.96	0.96-0.97	0.0001
**76-100th Percentile**	0.95	0.94-0.96	0.0001
**Primary Payer**			
**Private**	Reference		
**Medicaid**	1.2	1.2-1.3	0.0001
**Medicare**	1.0	1.0-1.0	0.1
**None**	0.9	0.8-0.9	0.0001
**Other**	1.4	1.4-1.5	0.0001
**Self-Pay**	1.5	1.5-1.5	0.0001
**Comorbidity Score**	1.2	1.2-1.2	0.0001
**Admission Day**			
**Weekday**	Reference		
**Weekend**	1.1	1.0-1.1	0.0001
**MDC**			
**Nervous System**	Reference		
**Alcohol/Drug Use**	0.1	0.1-0.1	0.0001
**Burns**	1.8	1.6-1.9	0.0001
**Circulatory**	0.5	0.5-0.5	0.0001
**Digestive**	0.5	0.5-0.5	0.0001
**Endocrine System**	0.4	0.4-0.4	0.0001
**Eyes**	0.1	0.1-0.1	0.0001
**Female Reproductive System**	0.4	0.4-0.5	0.0001
**Hepatobiliary**	0.7	0.7-0.7	0.0001
**HIV**	1.1	1.1-1.1	0.0001
**Immunologic**	0.4	0.4-0.4	0.0001
**Infectious**	3.3	3.2-3.3	0.0001
**Injuries/Poisons**	0.5	0.5-0.5	0.0001
**Kidneys**	0.6	0.5-0.6	0.0001
**Male Reproductive System**	0.3	0.3-0.3	0.0001
**Mental Health**	0.1	0.1-0.1	0.0001
**Musculoskeletal**	0.3	0.3-0.3	0.0001
**Myeloproliferative**	1.5	1.5-1.6	0.0001
**Neonatal**	1.4	1.4-1.4	0.0001
**Other Health Factors**	0.3	0.3-0.3	0.0001
**Otorhinolaryngology**	0.2	0.2-0.2	0.0001
**Pregnancy And Childbirth**	0.0	0.0-0.0	0.0001
**Pre-Major Diagnostic Category**	1.4	1.3-1.5	0.0001
**Respiratory**	1.2	1.2-1.2	0.0001
**Skin Or Breast**	0.2	0.2-0.2	0.0001
**Trauma**	5.1	5.0-5.3	0.0001
**Hospital Control**			
**Not-For-Profit, Nongovernment**	Reference		
**Government**	1.1	1.1-1.1	0.0001
**For-Profit, Investor Owned**	0.97	0.96-0.99	0.0001
**Region**			
**Northeast**	Reference		
**Midwest**	0.9	0.9-0.9	0.0001
**South**	0.9	0.9-0.9	0.0001
**West**	0.95	0.94-0.96	0.0001
**Urbanicity**	1.01	1.0-1.02	0.03
**Critical Access**			
**Yes**	0.8	0.8-0.8	0.0001
**Missing**	0.8	0.8-0.9	0.0001
**Rural Referral Center**			
**Yes**	1.1	0.1-1.1	0.0001
**Missing**	1.4	1.4-1.6	0.0001
**Networked**			
**Yes**	0.99	0.99-1.00	0.02
**Missing**	0.9	0.9-0.9	0.0001
**Medical Surgical Intensive Care**	1.0	1.0-1.0	0.5
**Hospice Program**	1.0	1.0-1.0	0.5
**Cardiac Intensive Care**	1.04	1.03-1.05	0.0001
**Pediatric Hospital**	0.97	0.96-0.99	0.0001
**Pediatric Intensive Care**	1.05	1.04-1.06	0.0001
**Neonatal Intensive Care Unit**	1.0	1.0-1.0	0.4
**Skilled Nursing Care**	1.04	1.03-1.05	0.0001
**JCAHO Accreditation**	1.04	1.02-1.05	0.0001
**ACS Cancer Program**	1.0	1.0-1.0	0.1
**Radiology, Photon Emission**	0.98	0.97-0.99	0.0001
**Radiology, CT Scan**	0.9	0.9-0.9	0.0001
**Radiology, PET Scan**	1.1	1.1-1.1	0.0001
**Medical Beds**			
**0-20**	Reference		
**21-74**	0.8	0.8-0.8	0.0001
**≥74**	0.8	0.8-0.8	0.0001
**Missing**	0.8	0.8-0.8	0.0001
**Total Hospital Beds**			
**0-53**	Reference		
**54-157**	1.0	1.0-1.0	0.3
**≥157**	1.1	1.0-1.1	0.0001
**Pediatric Beds**			
**0**	Reference		
**1-15**	1.0	1.0-1.0	0.1
**≥16**	0.1	1.0-1.1	0.0001
**Medical & Dental Trainees**			
**0**	Reference		
**1-26**	1.03	1.02-1.04	0.0001
**≥27**	1.05	1.04-1.07	0.0001
**Physicians/Hospital Bed**			
**0-0.007**	Reference		
**0.007-0.067**	0.9	0.9-0.9	0.0001
**≥0.067**	0.9	0.9-0.9	0.0001
**Nurses/Hospital Bed**			
**0-0.75**	Reference		
**0.75-1.359**	0.9	0.9-1.0	0.0001
**≥1.359**	0.9	0.9-0.9	0.0001

### Tests for interactions

We identified no interactions between staffing (registered nurse or physician) and admission day on mortality. However, we identified significant interactions for the presence of resident trainees. Mortality following a weekend admission to a hospital with the highest tertile of resident trainees was 17% higher (odds ratios 1.17 vs. 1.05) than hospitals with no resident trainees (p < 0.001) (Table 4) after adjusting for all covariates outlined in Table 3. This excess mortality following a weekend admission is significantly higher than the 5% increase observed between tertiles when admission occurs on a weekday (p-value for interaction < 0.001).

**Table 4:** Adjusted odds of mortality with test for interaction between full-time resident trainees and day of admission

	**Full time residents**		
**Day of Admission**	**0 (Referent)**	**1-26**	**≥27**
**Weekday**	1.0	1.03	1.05
**Weekend**	1.0	1.11	1.17

## Discussion

Using national all-payer discharge data, we confirmed significant differences in inpatient mortality as a function of day of admission. We also identified associations between staffing levels (both nursing and physician) and the outcome of mortality. However, the presence of resident physicians did not mitigate the effect of weekend day admission on mortality. Instead we found the reverse, a direct correlation between the presence of resident trainees and higher mortality. Most importantly, we identified interactions between admission day and the presence of resident trainees on the likelihood of inpatient mortality. These results may indicate that inadequate resident supervision by staff physicians during weekend patient care may adversely impact patient outcomes.

A prior study [21] did identify increased mortality at hospitals classified as “teaching” in the one state and with a smaller sample of medical diagnoses. However given the limited number of diagnoses and sample size in that study [21], we performed the study with a larger group of diagnoses, with nonelective admissions, and with a national sample of patients. In emergency settings, we hypothesized that trainees provide continuous on-site patient care, which is both invaluable and timely. Hospitals with residency training programs would thus harness the benefits of a team structure to care, endorsing multiple patient evaluations and re-evaluations and a care system focused on checks and balances. For example, in the setting of an intensive care unit, Poses and colleagues reported that the combined judgment of two junior or senior house officers was as good as that of the attending physician in managing the intensive care unit patient [22]. In a review by Kupersmith, teaching hospitals demonstrated better-quality measures, particularly measures of process, than did nonteaching hospitals [23]. The attention to detail inherent in a setting where trainees are present, general use of current medical literature to guide clinical decision making, and more frequent and thorough case reviews should contribute to a lower incidence of adverse occurrences [24]. In fact, improved outcomes have been demonstrated in some studies evaluating the role of residency programs. In a review of outcomes based on teaching status, lower risk-adjusted mortality was noted in major teaching hospitals for elderly patients with common conditions such as acute myocardial infarction, congestive heart failure, and pneumonia [25]. Contrary to these prior findings, our study of nonelective admissions to acute care hospitals revealed the opposite effect. It should be understood however, that our study was specific to nonelective admissions on the weekend alone.

In addition to the role of trainees on admission day outcomes, the difference in mortality following weekend admission as compared to weekdays was noted at hospitals without resident trainees as well. However, the mortality differences based on day of admission were particularly augmented at those hospitals in which residents train. We used tertiles for cutoffs of trainees to understand this effect because of the lack of data demonstrating any particular trainee presence as correlated with outcome. We noted that although mortality was higher on weekends whether or not a facility had a training program, that the mortality difference was substantially higher if the patient was admitted on a weekend to a facility with more resident trainees. The consistent elevation in mortality based on a weekend admission however implicate a more central structural or process measure, which is more fundamental than the role of trainees. It is likely that a common factor that is linked to residency training is critical to this admission day outcomes difference. This common factor may be related to supervision, provider staffing, care integration, or other specific provider factors. Our data did demonstrate higher mortality after weekend admission for patients in hospitals with fewer nurses and staff physicians, but this staffing finding is contrary to the resident trainee effect. Thus, although there is a value to more staff nurses [26], and physicians, more resident trainees seem to have the opposite effect on mortality. This conflicting finding leads us to more questions than answers regarding the role of staffing on healthcare outcomes.

Our study has limitations based on the data used for analysis. Although the administrative data used in our study are population based, there is the potential for information and misclassification bias. It is unlikely that mortality or day of admission were improperly abstracted from the medical record; however, staffing levels and the presence of trainees may not be as rigorously documented. The data are limited to inpatient mortality and there is not assumption that the mortality trend persists beyond the inpatient setting. It should be understood that our data do not tie a direct link between the care provided by a trainee and the outcome of mortality. Our data only demonstrate that the increased mortality noted when patients are admitted on a weekend is worse when a facility has resident trainees. In addition to this concern, it is possible that patients admitted during the weekend have more comorbidities or potentially more severe illnesses at hospitals with resident trainees. Although we have adjusted for comorbidity, an assessment of disease severity at presentation is not possible with the available data. However, others have not identified significant differences in healthcare resource groupings indicating high comorbidity or complications at National Health Service hospitals in England across admission day [2]. Thus, despite these limitations, the strength of our study is in the large population studied implying that the results are representative and generalizable to the nation.

## Conclusions

In conclusion, our data indicate that the role of staffing and resident trainees is an important area of focus for patient safety. Our study does not argue against a positive benefit of resident trainees to the sponsoring institution, the local community, affiliated academic health center, and the greater community [24]. Rather, our data suggest that the role of resident supervision may be an important target for quality weekend medical care. Although a causal link between supervision and outcome can not be identified in this study, our data do demonstrate that hospitals with many trainees have the highest mortality burden following a weekend admission. An assessment of rounding practices, trainee-directed procedures, and/or call routines of supervising physicians would help answer questions raised by our study. Following these assessments, strategies can be developed and implemented to standardize care across admission day.

## Competing interests

Dr. Schoetz is the Chair of Medical Education at Lahey Hospital and Medical Center.

## Authors’ contributions

Conception and Design: RR, JN, Acquisition of Data: RR, Analysis and Interpretation of Data: RR, JN, DJS, TER, PLR, PWM, Drafting the Article: RR, JN, Critical Revision: RR, JN, DJS, TER, PLR, PWM, Final Manuscript Approval: RR, JN, DJS, TER, PLR, PWM. All authors read and approved the final manuscript.

## Pre-publication history

The pre-publication history for this paper can be accessed here:

http://www.biomedcentral.com/1472-6920/14/4/prepub
